# Early Pro-inflammatory Microglia Activation After Inflammation-Sensitized Hypoxic-Ischemic Brain Injury in Neonatal Rats

**DOI:** 10.3389/fncel.2019.00237

**Published:** 2019-05-24

**Authors:** Meray Serdar, Karina Kempe, Mandana Rizazad, Josephine Herz, Ivo Bendix, Ursula Felderhoff-Müser, Hemmen Sabir

**Affiliations:** ^1^Department of Pediatrics I, Neonatology and Experimental Perinatal Neuroscience, University Hospital Essen, University Duisburg-Essen, Essen, Germany; ^2^Department of General Pediatrics, Neonatology and Pediatric Cardiology, University Children’s Hospital Düsseldorf, Medical Faculty, Heinrich Heine University, Düsseldorf, Germany; ^3^Department of Neonatology and Pediatric Intensive Care, Children’s Hospital, University of Bonn, Bonn, Germany

**Keywords:** newborn, HIE, brain, infection, inflammation, microglia

## Abstract

**Background:** Perinatal asphyxia, leading to neonatal encephalopathy, is one of the leading causes for child mortality and long-term morbidities. Neonatal encephalopathy rates are significantly increased in newborns with perinatal infection. Therapeutic hypothermia is only neuroprotective in 50% of cooled asphyxiated newborns. As shown experimentally, cooling has failed to be neuroprotective after inflammation-sensitized hypoxic ischemic (HI) brain injury. Microglia are thought to be key players after inflammation-sensitized HI brain injury. We performed this study investigating early microglia phenotype polarization in our newborn animal model of inflammation-sensitized HI brain injury, better understanding the underlying pathophysiological processes.

**Methods:** Seven days old Wistar rat pups were injected with either vehicle (NaCl 0.9%) or E. coli lipopolysaccharide (LPS), followed by left carotid ligation combined with global hypoxia inducing a mild unilateral hypoxic-ischemic injury. Pups were randomized to (1) Sham group (*n* = 41), (2) LPS only group (*n* = 37), (3) Veh/HI group (*n* = 56), and (4) LPS/HI group (*n* = 79). On postnatal days 8 and 14 gene-expression analysis or immunohistochemistry was performed describing early microglia polarization in our model.

**Results:** We confirmed that LPS pre-sensitization significantly increases brain area loss and induced microglia activation and neuronal injury after mild hypoxia-ischemia. Additionally, we show that microglia upregulate pro-inflammatory genes involving *NLRP-3* inflammasome gene expression 24 h after inflammation-sensitized hypoxic-ischemic brain injury.

**Conclusion:** These results demonstrate that microglia are early key mediators of the inflammatory response following inflammation-sensitized HI brain injury and that they polarize into a predominant pro-inflammatory phenotype 24 h post HI. This may lead to new treatment options altering microglia phenotype polarization early after HI brain injury.

## Introduction

Perinatal asphyxia is one of the leading causes of neonatal mortality and long-term mental and motor disabilities, including cerebral palsy (Jacobs et al., 2013). Between 2 and 4 of 1000 term newborns in the western world suffer from birth asphyxia, whereas globally the incidence is described to be much higher (Jacobs et al., 2013; United Nations Report, 2015). Perinatal asphyxia may lead to neonatal encephalopathy (NE), most likely due to hypoxia-ischemia (HI). Currently therapeutic hypothermia (TH) is the standard treatment for hypoxic-ischemic encephalopathy (HIE), however, only 50% of cooled asphyxiated newborns benefit from cooling treatment (Jacobs et al., 2013). Early identification of “non-responders" to cooling therapy is not feasible yet, as specific and robust biomarkers are lacking and early identification of non-responders is yet not possible. In low- and middle-income countries, where NE rates are significantly higher, the introduction of TH was unsuccessful, increasing mortality (Robertson et al., 2011). Perinatal infection is a well-recognized risk factor for cerebral palsy, long-term disability and mortality in term newborns (Grether and Nelson, 1997). Recently, it has been shown that neonatal infection rates in asphyxiated newborns are significantly higher, compared to the general population (Tann et al., 2017). Whether TH is neuroprotective in these newborns remains unknown. We have previously shown that TH is not neuroprotective in our established animal model of inflammation-sensitized hypoxic-ischemic brain injury (Osredkar et al., 2013, 2015). However, the underlying mechanisms remained unclear.

Microglia, the tissue-resident macrophages of the central nervous system (CNS), are responsible for combating infection, clearing cellular debris, and maintaining tissue homeostasis (Pierre et al., 2017; Lenz and Nelson, 2018). Within minutes following an injurious insult, such as hypoxia, ischemia, infection and trauma, microglia become activated, changing their gene expression profile (Ohsawa and Kohsaka, 2011; Lenz and Nelson, 2018). We have previously shown that microglia cells are significantly upregulated in our inflammation-sensitized model of HI brain injury (Osredkar et al., 2015; Falck et al., 2018). However, we have not further investigated microglial polarization yet. Activated microglia are capable to polarize into different phenotypic categories (Lenz and Nelson, 2018). The M1 microglia, or classical activated microglia phenotype is associated with an increased production of pro-inflammatory cytokines and chemokines (Chhor et al., 2013; Cunha et al., 2016). The alternative anti-inflammatory M2 phenotype is less aggressive to the neuronal tissue, promoting tissue repair, phagocytosis of protein aggregates and cell debris (Wang et al., 2014).

The *nucleotide-binding domain, leucine-rich repeat protein (NLRP)-3* inflammasome is highly involved in neonatal brain injury either due to LPS or hypoxia-ischemia (Hedtjarn et al., 2002; Gustin et al., 2015). Regarding our inflammation-sensitized HI model, no data are available on *NLRP3* activity yet. *NLRP3* is responsible for the cleavage of *interleukin IL-18* and *IL-1beta* from its preforms. As shown the vulnerability of the neonatal brain to LPS or hypoxia-ischemia is *IL-18* and *IL-1beta* dependent predominantly leading to microglia activation (Gustin et al., 2015).

The mechanisms responsible for microglia phenotype regulation in the newborn CNS are poorly understood. The interaction of M1/M2 microglia does not seem to follow strict differentiation, but to be a complex time dependent continuum, which may be altered due to its actual needs (Wang et al., 2014; Mallard et al., 2018). Following neonatal HI brain injury, it has been shown that M1/M2 specific genes are upregulated in a time dependent manner, with early M1 response and delayed M2 response possibly regulating the pro-inflammatory, neurotoxic cascade (Hellstrom Erkenstam et al., 2016). Furthermore, it has been successfully shown that shifting the microglia phenotype toward an M2 phenotype, using TH, is neuroprotective in an adult traumatic brain injury animal model (Truettner et al., 2016). In our neonatal model of inflammation-sensitized HI the time dependent microglial phenotype expression pattern has yet not been investigated. As TH is not neuroprotective in our experimental setup, understanding microglia phenotype polarization might help to develop new additional treatment options. Therefore, we examined early M1/M2 marker gene expression in different brain regions (hippocampus and cortex) and in *ex vivo* isolated microglia cells in our newborn animal model of inflammation-sensitized HI brain injury.

## Materials and Methods

### Animals and Experimental Procedure

All animal experiments were performed in accordance to the Animal Research: Reporting of *in vivo* Experiments (ARRIVE) guidelines with government approval by the State Agency for Nature, Environment and Consumer Protection North Rhine-Westphalia, Germany. We used 7-day old (P7) Wistar rat pups of both genders in all our experiments. All pups were kept at the central animal laboratory of the University Hospital Essen, Germany with a 12:12 h dark:light cycle at an environmental temperature of 21°C with food and water *ad libitum*. As previously described, all animals were randomized across litter, sex, and weight before the experiments commenced and all following experiments and analysis were performed by observers blinded to the different treatments (Osredkar et al., 2013, 2015).

A total of 223 P7 rat pups (*n* = 108 female; *n* = 115 male) from 21 litters were used in this study. Temperature during handling and experimental procedures was monitored in “sentinel” rat pups (*n* = 10) not further allocated to the different treatment groups. All rat pups were kept on a servo-controlled mat (CritiCool, MTRE, Yavne, Israel) during separation from their dams, controlled by the sentinel pup via a rectal temperature probe (IT-21, Physitemp Instruments, Clifton, NJ, United States), continuously maintaining nesting temperature of P7 rat pups (Wood et al., 2016) or treatment temperatures during experiments (see below). Forty-one rats underwent sham surgery (Sham group) and 37 rats received a single i.p. injection of lipopolysaccharide (LPS) solution (*Escherichia coli* O55:B5, Sigma; 0.1 mg/kg) followed by sham surgery (LPS group). The remaining animals (*n* = 135) were exposed to our inflammation-sensitized model of hypoxic-ischemic brain injury as previously described (Osredkar et al., 2013, 2015). In brief, at the start of every experiment, animals were injected according to randomization with either a single intraperitoneal (i.p.) injection of vehicle solution (0.9% NaCl; Veh/HI group, *n* = 56) or LPS solution (Escherichia coli O55:B5, Sigma; 0.1 mg/kg; LPS/HI group, *n* = 79). After a 4 h delay, whilst animals were kept with their dams, animals were exposed to HI as described. Under general isoflurane anesthesia, the left common carotid artery was ligated and cut. Within 3 h the pups were subjected to 8% O_2_ for 50 min at a rectal temperature (T_rectal_) of 36°C, resulting in mild HI injury (Osredkar et al., 2013, 2015). Immediately after the HI insult, pups were kept at T_rectal_ of 37.0°C for 5 h, representing the normothermia treatment group in our previous experiments (Sabir et al., 2012; Osredkar et al., 2013, 2015). After the treatment period, pups were immediately returned to their dam.

For histological analysis and determination of brain area loss pups were sacrificed at P14. Rats were transcardially perfused with phosphate-buffered saline (PBS) followed by 4% paraformaldehyde (Sigma-Aldrich). Brains were post-fixed in 4% paraformaldehyde overnight at 4°C and embedded in paraffin. For mRNA and microglia analyses pups were transcardially perfused with PBS. For mRNA analysis, different brain regions (hippocampus and cortex) were prepared, using a standard matrix for uniformity (ASI instruments Inc., Warren, MI, United States) and immediately snap-frozen in liquid nitrogen.

### Area Measurement

In total 52 rat pups were used for area loss analysis [*n* = 13 in group (1), *n* = 10 in group (2), *n* = 17 in group (3), *n* = 12 in group (4)]. The embedded brains were cut in 10 μm coronal sections. Sections were stained with cresyl-violet. Brain area loss was determined by measurement of intact areas in ipsilateral and contralateral hemispheres of two sections, from two neighboring blocks (-3.72 ± 0.7 mm from Bregma) at a distance of 50 μm using ImageJ software (ImageJ, version 1.46r, National Institutes of Health, Bethesda, MD, United States). Tissue loss was determined by comparison with contralateral areas according to the following equation: 1- (area ratio (left vs. right)) × 100 (Sabir et al., 2012) and mean values of the two analyzed sections were calculated.

### Immunohistochemistry

Immunohistochemistry was performed as previously described (Serdar et al., 2016). After deparaffinization, 10 μm coronal sections (-3.72 ± 0.7 mm from Bregma) were rehydrated. Antigen retrieval was performed in a pre-heated 10 mM sodium-citrate buffer (pH 6.0) for 30 min. After blocking with 1% bovine serum albumin and 0.3% cold fish skin gelatine in 0.1% Tween-20 TBS (all Sigma–Aldrich, Germany), slides were incubated with primary antibodies overnight at 4°C followed by appropriate secondary antibody incubation for 1h at room temperature. Sections were counterstained with 4,6-diamidino-2-phenylindole (DAPI) (1 μg/ml, Invitrogen, Germany). Microglia activation was detected by Iba1 (1:1000, rabbit polyclonal anti-Iba1, Wako, Germany) staining on sections of P14 rat pups. At the same time point neurons were evaluated using the marker NeuN (1:200, polyclonal rabbit anti-NeuN, Millipore, Germany). Two regions of interest (ROI, each 45,500 μm^2^) in the hippocampus (CA1 and CA2 region) and cortex of contra- and ipsilateral brain hemispheres were visualized by fluorescence microscopy (Axioplan; Zeiss, Germany) connected to a CCD camera (Axioplan, Zeiss, Germany). The area of positive staining of the ipsilateral side was determined by using Image J software and was normalized to the contralateral side (Reinboth et al., 2016). There were no differences in staining of the contralateral side.

### Magnetic Activated Cell Sorting (MACS) of CD11 b/c Positive Microglia

To analyze the different alterations in phenotype polarization of microglia, we specifically isolated CD11 b/c positive microglia *ex vivo* from rat brains 24 h after hypoxia. In total 32 rat pups were used [*n* = 8 in group (1), *n* = 8 in group (2), *n* = 8 in group (3), *n* = 8 in group (4)]. At first, pups were perfused with PBS. In groups (1) and (2) full brains (ipsi-/contralateral hemispheres) were used for analysis, while in group (3) and (4) ipsilateral hemispheres were pooled to get a workable concentration of microglia. A Neural tissue dissociation kit (Miltenyi Biotech) was used for mechanical and enzymatic dissociation of brain tissues. Myelin-removal was performed following distributors instructions before MACS. For MACS the obtained cell mixtures were washed with MACS buffer (PBS containing 0.5% BSA) and incubated with anti-CD11b/c coupled microbeads (Miltenyi Biotech) followed by magnetic separation on MS columns of the MiniMACS magnetic separation kit (Miltenyi Biotech, Germany). The cell mixture was then passed through the column placed in MiniMACS magnets followed by a series of three washes (500 μl each). The total effluent was collected as the negative fraction. After removal of the column CD11 b/c positive microglia were eluted in a volume of 1 ml MACS buffer. To investigate the purity of the magnetically separated cells, the positive eluate was analyzed via immunocytochemistry through Iba1 staining (data not shown).

### Real-Time PCR

RNA was generated from ipsilateral regions through the classic phase extraction method, using TRIzol and Chloroform. First strand complementary DNA was synthesized using 1 or 4 μg of total RNA and TaqMan reverse transcription reagents (Applied Biosystems/Thermo Fisher Scientific, United States). PCR amplification was performed in 96 well optical reaction plates for 40 cycles with each cycle at 94°C for 15 s and 60°C for 1 min using the StepOnePlus Real Time PCR system (Applied Biosystems/Thermo Fisher Scientific, United States). Analysis was performed 24 h post HI using the hippocampus and cortex of the ipsilateral hemispheres in our 4 pre-defined groups [*n* = 7 in group (1), *n* = 7 in group (2), *n* = 12 in group (3), *n* = 12 in group (4)]. The PCR products of pro- and anti-inflammatory cytokines, associated with classical or alternative microglial activation, were quantified by fluorogenic reporter oligonucleotide probes. Pro-inflammatory markers used in this study are: *Interleukin IL-1beta* (Rn00580432_m1; life technologies, Germany), *IL-6* (Rn01410330_m1; Life Technologies, Germany), *inducible nitric oxide synthase (iNOS)* (Rn00561646_m1; Life Technologies, Germany) and *IL12* (Rn00584538_m1; Life Technologies, Germany). Anti-inflammatory markers used in this study are *transforming growth factor (TGF)-beta* (Rn00572010_m1; Life Technologies, Germany), *Arginase-1* (Rn00691090_m1; Life Technologies, Germany) and *cluster of differentiation (CD)-206* (Rn01487342_m1; Life Technologies, Germany). Additionally, we analyzed gene expression of the inflammasome *cryopyrin (Nlrp3)* (Rn04244620_m1; Life Technologies, Germany). *Beta-2-microtubulin (B2M)* was used as housekeeping gene (Rn00560865_m1; Life Technologies, Germany). Results were normalized to the ipsilateral hemispheres of the sham group.

To further analyze microglia polarization in this model, the same pro- and anti-inflammatory marker gene expression was analyzed in the previous described CD11 b/c positive microglia cells.

Generally, RealTime PCR and detection were performed in duplicates; measurements were repeated two times for each sample. Target gene expression was quantified according to the 2^ΔΔCT^ method (Livak and Schmittgen, 2001).

### Statistical Analysis

Graphical data are presented as median values with 95% confidence intervals or boxplots including the 25% and the 75% percentile. Data were analyzed using GraphPad Prism 6 (GraphPad Software, United States). Non-parametric statistics were applied. The Kruskal–Wallis test was used for comparisons across multiple treatment groups, and the Bonferroni *post hoc* test was used for 2-group comparisons. *p*-values less than 0.05 were considered as statistically significant.

## Results

In total 10 experiments were performed using a 4-group design: (1) Sham group, (2) LPS group, (3) Veh/HI group and (4) LPS/HI group. Out of the 213 animals used in our 4-group design, mortality was highest in the LPS/HI group. In total 40 animals died during HI [three animals from group (2), two animals from group (3), 35 animals from group (4)], leaving 173 rat pups for further analysis. The high mortality in the LPS/HI group has been expected and reported by us before (Osredkar et al., 2013, 2015) and we accounted this during group randomization.

### LPS Pre-sensitization Increases Brain Area Loss and Induces Microglia Activation and Neuronal Injury After Mild Hypoxia-Ischemia

**FIGURE 1:**
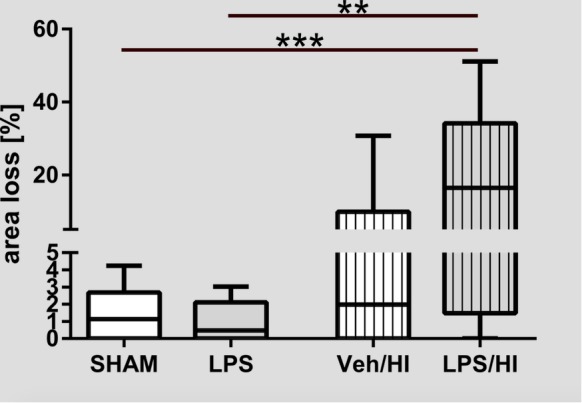
Brain area loss 1 week after inflammation-sensitized hypoxic-ischemic brain injury. We found increased brain area loss of ipsilateral brain hemispheres in the LPS/HI group compared to the other treatment groups. In the Veh/HI group injury was mild, representing the mild character of our hypoxic-ischemic insult. Group sizes: Sham *n* = 13, LPS *n* = 10, Veh/HI *n* = 17, LPS/HI *n* = 12; ^∗∗^*p* < 0.01, ^∗∗∗^*p* < 0.001.

As shown in Figure 1, pre-sensitization with LPS 4 h prior to a mild HI insult, increases brain area loss compared to a mild HI insult alone at P14. Median area loss was 1.1% (0.5 – 2.3) in group (1), 0.5% (0 – 2.2) in group (2), 5.9% (1.4 – 10.5) in group (3) and 19.3% (7.5 – 31.2) in group (4), respectively. The low area loss in group (3) indicates the mild nature of our hypoxic-ischemic insult, following 50 min of hypoxia at T_rectal_ of 36°C. This mild insult was intended, as LPS pre-sensitization combined with a moderate to severe insult would result in almost 100% mortality in the LPS/HI group, as previously shown by ourselves and others before (Eklind et al., 2001; Osredkar et al., 2013, 2015).

**FIGURE 2:**
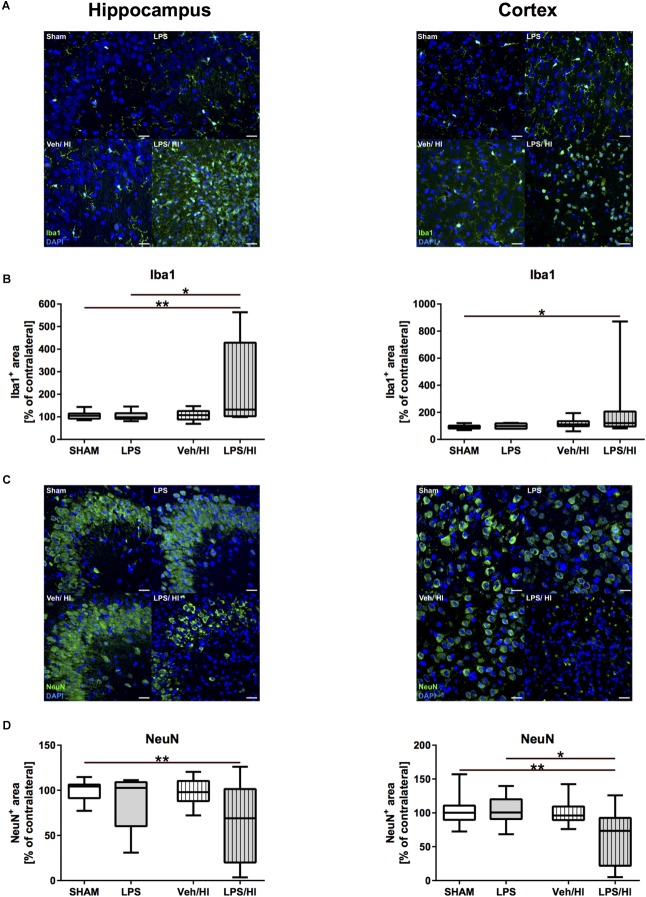
Microglia and neuronal density 1 week after inflammation-sensitized hypoxic-ischemic brain injury. **(A)** Representative Iba1 staining images from the hippocampus and cortex. **(B)** There was a significantly increased Iba 1 staining in the hippocampus and cortex of rat pups of the LPS/HI group compared to the sham (hippocampus and cortex) and LPS group (cortex). **(C)** Representative NeuN staining images of the hippocampus and cortex. **(D)** Neuronal staining was significantly reduced in the hippocampus and cortex of the LPS/HI group compared to sham-operated rats (hippocampus and cortex) and LPS-treated animals (cortex). Group sizes: Sham *n* = 13, LPS *n* = 9, Veh/HI *n* = 17, LPS/HI *n* = 12; ^∗^*p* < 0.05, ^∗∗^*p* < 0.01.

Immunohistochemistry showed that Iba1 staining was significantly increased in the hippocampus and cortex of animals from the LPS/HI group compared to the sham (hippocampus: *p* = 0.0012; cortex: *p* = 0.0390) and LPS group (hippocampus: *p* = 0.0103) (Figure 2A,B). In addition, we found that neuronal density was significantly decreased in the hippocampus and cortex of animals from the LPS/HI group compared to the sham (hippocampus: *p* = 0.0086; cortex: *p* = 0.0068) and LPS group (cortex: *p* = 0.0373) (Figure 2C,D). These findings demonstrate that pre-sensitization with a non-injurious dose of LPS significantly increases brain damage in a mild HI brain injury model and underlie the important role of microglia in our established inflammation-sensitized hypoxic-ischemic brain injury model, leading to further analyses.

**FIGURE 3:**
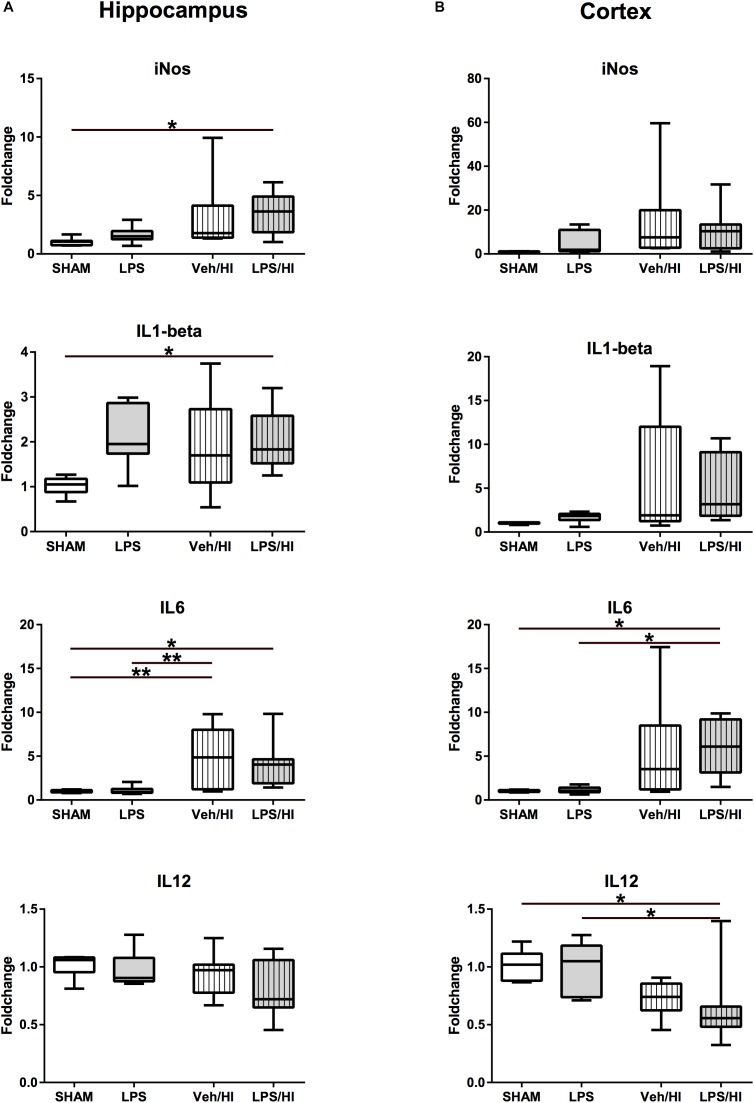
Pro-inflammatory cytokine expression 24 h after inflammation-sensitized hypoxic-ischemic brain injury. **(A)** In the hippocampus pro-inflammatory genes of *iNOS, IL-1beta* and *IL-6* were significantly upregulated in animals pre-sensitized with LPS undergoing a mild HI injury. **(B)** In the cortex, *IL-6* was significantly upregulated, whilst *IL-12* was downregulated after inflammation-sensitized hypoxic-ischemic brain injury. Group sizes: Sham *n* = 7, LPS *n* = 7, Veh/HI *n* = 12, LPS/HI *n* = 12; ^∗^*p* < 0.05, ^∗∗^*p* < 0.01.

### Early Pro-inflammatory Gene Expression Following Inflammation-Sensitized Hypoxic-Ischemic Brain Injury

To further determine early inflammatory responses in our model, we assessed gene expression profiles associated with M1/M2 microglial polarization in total brain lysates of the hippocampus and the cortex. We determined a significantly upregulated gene expression of pro-inflammatory molecules, e.g., *iNOS* (*p* = 0.0483), *IL-1beta* (*p* = 0.049), and *IL-6* (*p* = 0.0492) in the hippocampus of the LPS/HI group compared to sham controls (Figure 3A). Animals from the Veh/HI group only presented with mild upregulation of pro-inflammatory genes [*IL-6* in hippocampus (*p* = 0.0024, Figure 3A)], mainly due to the aimed mild injury in this group. Additionally, *IL-6* was significantly upregulated (*p* = 0.0191), while *IL-12* was significantly downregulated (*p* = 0.0490) (Figure 3B) in the cortex of animals from the LPS/HI group.

**FIGURE 4:**
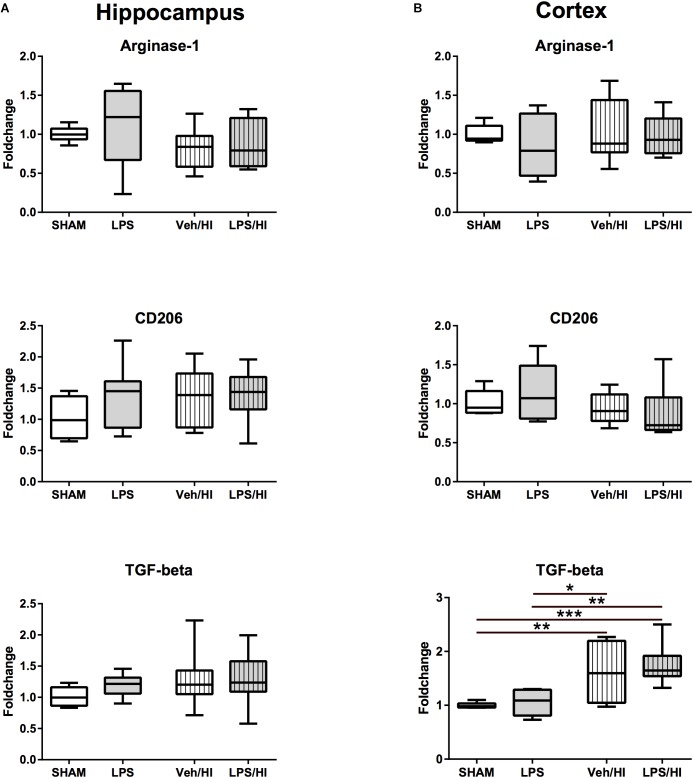
Anti-inflammatory cytokine expression 24 h after inflammation-sensitized hypoxic-ischemic brain injury. **(A)** There was no significant difference for the further analyzed cytokines in the hippocampus 24 h after our experiments. **(B)** There was a significant upregulation of *TGF-beta* in the cortex of animals from the Veh/HI and LPS/HI groups compared to the Sham and LPS groups. Group sizes: Sham *n* = 7, LPS *n* = 7, Veh/HI *n* = 12, LPS/HI *n* = 12; ^∗^*p* < 0.05, ^∗∗^*p* < 0.01, ^∗∗∗^*p* < 0.001.

Besides a significant increase in *TGF-beta* expression in the cortex of the VEH/HI and LPS/HI group compared to the Sham and LPS group (*p* < 0.05, Figure 4B), M2 associated anti-inflammatory gene expression was not significantly modulated by the different treatments at the analyzed time point (Figure 4).

Overall, these results show an early significant M1 associated pro-inflammatory gene expression in the hippocampus and cortex predominately in rats of the LPS/HI group compared to the Sham- and LPS/Veh group.

### Microglia Reveal Early Pro-inflammatory Gene Expression Following Inflammation-Sensitized Hypoxic-Ischemic Brain Injury

**FIGURE 5:**
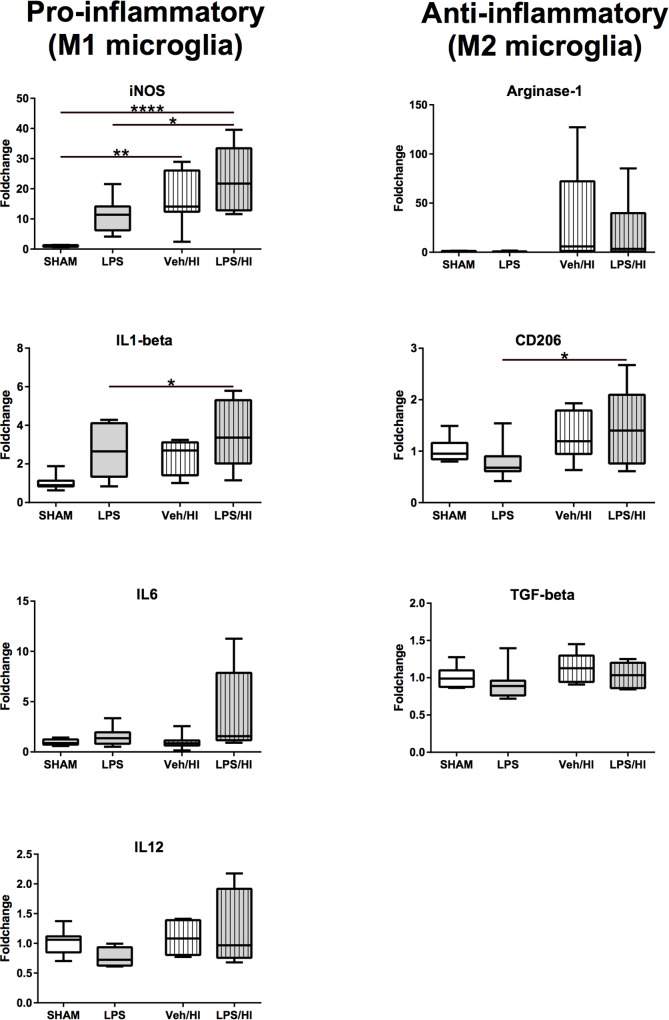
Gene-expression of CD11b/c microglia 24 h after inflammation-sensitized hypoxic-ischemic brain injury. Gene expression of pro-inflammatory genes for *iNOS* and *IL-1beta* was significantly upregulated in microglia from the LPS/HI group compared to Sham. Additionally, gene expression of the anti-inflammatory gene for *CD206* was significantly upregulated in microglia from the LPS/HI group. Group sizes: Sham *n* = 8, LPS *n* = 8, Veh/HI *n* = 8, LPS/HI *n* = 8; ^∗^*p* < 0.05, ^∗∗^*p* < 0.01, ^∗∗∗∗^*p* < 0.0001.

After we found that pro-inflammatory gene expression was upregulated in total brain lysates of the LPS/HI group, we specifically wanted to analyze the role of CD11b/c microglia, as microglia are supposed to be a major source of pro-inflammatory gene expression (Hellstrom Erkenstam et al., 2016). Therefore, we sorted CD11b/c positive microglia via magnetic activated cell sorting 24 h post HI. Similarly, as in total brain lysates, we observed a significant increase in M1 associated pro-inflammatory gene expression for *iNOS* (*p* < 0.0001) and *IL-1beta* (*p* = 0.0029) in microglia of the LPS/HI group compared to the LPS and Sham group (Figure 5). *iNOS* was also significantly upregulated in the Veh/HI group, compared to the sham group (*p* = 0.0015). In addition, a significant upregulation of *CD206* (*p* = 0.031) was detected in microglia of the LPS/HI group, compared to the LPS group (Figure 5).

These results underlie the role of pro-inflammatory M1 microglia polarization in animals pre-sensitized with LPS prior to hypoxia-ischemia, compared to animals from the Veh/HI and other groups in this specific combined animal model.

### *NLRP3* Inflammasome Activation After Inflammation-Sensitized Hypoxic-Ischemic Brain Injury

**FIGURE 6:**
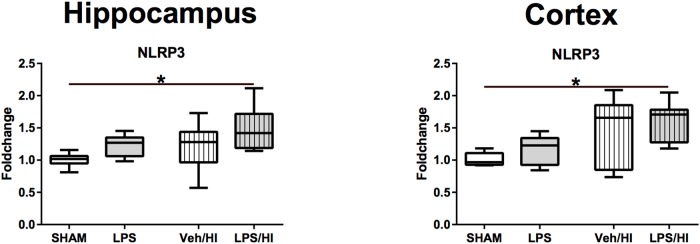
Gene expression of *NLRP3* 24 h after inflammation-sensitized hypoxic-ischemic brain injury. Gene expression of *NLRP3* was highest in the LPS/HI group in both analyzed brain regions and significantly increased compared to the Sham group in the hippocampus and cortex. Group sizes: Sham *n* = 7, LPS *n* = 7, Veh/HI *n* = 12, LPS/HI *n* = 12; ^∗^*p* < 0.05.

As *NLRP3* is responsible for the cleavage of *interleukin IL-18* and *IL-1beta* from its preforms, leading to microglia activation, we last analyzed *NLRP3* gene expression in our model. We found that 24 h post HI, *NLRP3* gene expression is significantly upregulated in the hippocampus (*p* = 0.0060) and cortex (*p* = 0.0081) of rats from the LPS/HI group compared to the Sham group (Figure 6).

## Discussion

The present study confirms that pre-exposure with a non-injurious dose of LPS followed by mild hypoxic-ischemic brain injury exacerbates brain injury in 7 days old rats. The mechanism is not fully understood. We now show that microglia gene expression is polarized into a M1, pro-inflammatory phenotype 24 h following inflammation-sensitized hypoxic-ischemic brain injury. Additionally, we highlight the involvement of the *NLRP3* inflammasome in the inflammatory process, probably activating *IL-1beta* in rats pre-exposed with LPS and undergoing our HI brain injury model. These results demonstrate that microglia are early key mediators of the inflammatory response following inflammation-sensitized HI brain injury, polarizing into a predominant pro-inflammatory (M1) phenotype 24 h post HI.

We have previously described the combined animal model of inflammation and HI brain injury by modification of the classical Rice-Vannucci rat model of HI brain injury followed by post-insult normothermia or hypothermia (Osredkar et al., 2013, 2015; Falck et al., 2018). The Rice-Vannucci HI rat model has been used for over three decades to describe and assess newborn brain injury, leading to translational clinical trials and the establishment of therapeutic hypothermia (TH) to reduce mortality and morbidities following perinatal asphyxia (Rice et al., 1981). However, clinically ∼50% of all cooled newborns from these large randomized controlled trials did not benefit from cooling therapy (Jacobs et al., 2013). Early identification of these non-responders is yet not possible.

Recently, it has been shown that neonatal infection rates in asphyxiated newborns are much higher, compared to the general population and that perinatal infection contributes to perinatal asphyxia (Tann et al., 2016, 2017). Whether TH is neuroprotective in these newborns remains unknown. A single-center study reported that TH was not neuroprotective in asphyxiated newborns with proven bacterial chorioamnionitis, despite antibiotic treatment (Wintermark et al., 2010). Recently, a retrospective analysis from the Netherlands and Belgium reported that outcome in newborns with perinatal asphyxia and proven culture-positive sepsis did not significantly differ from outcome of infants with perinatal asphyxia without sepsis (Hakobyan et al., 2018). However, the number of infants with culture-positive sepsis involved in both studies was very low.

We have previously shown that TH is not beneficial in our animal model of inflammation-sensitized HI brain injury and that microglia cells are activated in our injury model (positive Iba1 staining) (Osredkar et al., 2013, 2015). Before new treatment interventions can be explored and tested, we need to understand the underlying pathophysiology, leading to the failure of the established neuroprotective treatment (therapeutic hypothermia). In the current study, we aimed to specifically investigate the underlying early pathophysiological processes in our model of LPS-sensitized HI brain injury, focusing on the early polarization of microglia and to describe their role in sensitizing the brain to a higher degree of injury.

M1 microglia produce a large number of pro-inflammatory cytokines (e.g., *IL-6, IL-1beta, IL-12*), chemokines, reactive oxygen species and *iNOS* (Cunha et al., 2016; Hellstrom Erkenstam et al., 2016). These could potentially be used as early reliable biomarkers after HI or LPS exposition, discriminating between HI or infection-sensitized HI. Activated microglia have been found in the gray and white matter following HI. However, this has so far not been analyzed in a combined neonatal model of inflammation and hypoxia-ischemia. We show here that rats sensitized with LPS prior to HI brain injury reveal most pronounced changes/increases in the amount of activated microglia, assessed by total Iba-1 expression in the hippocampus and cortex following LPS/HI. This was accompanied by a significantly increased expression of pro-inflammatory cytokines and *iNOS*.

As the determined increased pro-inflammatory gene expression might also be due to reactive astrogliosis, we specifically sorted microglia cells, proving their specific role in our model. We confirmed that microglia associated genes are significantly upregulated in rats following LPS pre-exposure before HI brain injury compared to the other treatment groups and that microglia early polarize into a pro-inflammatory phenotype, leading to brain injury and neuronal loss at P14. As previously described, the interaction of M1/M2 microglia is a time dependent process (Hellstrom Erkenstam et al., 2016), which may be altered due to its actual needs, potentially leading to novel treatment options. This has successfully been shown in an experimental adult traumatic brain injury model, where M1/M2 phenotype polarization could be externally altered by cooling therapy, stimulating the polarization into the anti-inflammatory M2 phenotype resulting in neuroprotection (Truettner et al., 2016). As we have only analyzed one specific time point yet, further analyzing different time points is crucial. This will help us to describe the time-dependent shift of the M1 microglia, into the anti-inflammatory M2 microglia in our specific injury model. Most importantly, we have to identify the regulators of the presumed M1/M2 activation in our model. Despite hypoxia-ischemia, LPS exposure to the neonatal brain directly activates microglia (Mallard et al., 2018). Toll-like receptors (TLRs) are tightly linked to the innate immune response in microglia (Nair-Gupta et al., 2014). Microglia express a large repertoire of TLRs, allowing them to react to different pathogens. LPS primarily binds to TLR-4 (Feezor et al., 2003). Stimulation of TLR-4 triggers the translocation of *nuclear factor kappa B (NF-kappaB)* into the nucleus, expressing proinflammatory genes in microglia, involving the activation of the inflammasome (O’Callaghan et al., 2014; Cunha et al., 2016). The inflammasome is a *caspase-1* activating multiprotein that results from oligomerization of inactive monomeric proteins from the *NLRP* family. The most intensively studied inflammasome is *NLRP3*. *NLRP3* has been shown to be involved in many neurological diseases in adults, such as multiple sclerosis, Alzheimer’s and Parkinson’s disease (Strowig et al., 2012). After being activated by *NLRP3, IL-18* and *IL-1beta* are activated from their preforms. As shown here, the vulnerability of the neonatal brain to LPS and HI is also attributed to *IL-1beta*. We also show that *NLRP3* gene expression is highest and significantly upregulated in rats being exposed to LPS before undergoing our mild HI insult. This might be one involved regulatory pathway, explaining how LPS pre-exposure significantly increases the vulnerability of the newborn brain to a mild hypoxic-ischemic event.

In our study, we observed an upregulation of the anti-inflammatory cytokine *TGF-beta* in animals from the LPS/HI group. At first sight, these results seem controversial. However, it has been shown that *TGF-beta* is a pleiotropic cytokine, with potent regulatory and inflammatory activity (Sanjabi et al., 2009). In the presence of *IL-6, TGF-beta* induces the differentiation of specific T-cells (TH17), promoting an inflammatory response (Qin et al., 2009). Transferred to our findings, it might be possible that the observed upregulation of *IL-6* in the hippocampus induces an overexpression of the cytokine *TGF-beta* triggering the inflammatory process through the differentiation of TH17 cells, which migrated into the brain after the acute brain injury in our model. To verify this hypothesis, there is an urgent need to analyze the activation, migration and differentiation of leukocytes in our model. On the other hand, it has been shown that peripheral T-cell depletion exacerbates brain injury in a neonatal rodent model of HI brain injury (Herz et al., 2018). These findings underlie the importance of further investigating the role of different T cells subsets in newborn brain injury models, as there is a clear gap in knowledge about T-cell differentiation and -function in newborn brain injury models.

Furthermore, we observed a significant upregulation of *CD206* gene expression in microglia of the LPS/HI group. Conversely, *CD206* is thought to be associated with the M2, anti-inflammatory microglia phenotype. As described in detail by Hellstrom Erkenstam et al. (2016) this may underlie the complexity of the inflammatory responses and cascades using *in vivo* injury models and stresses the question whether using a simple M1/M2 phenotype theory is inadequate to describe the complex concept of distinct inflammatory cell phenotypes in the brain.

There are limitations to our study. First, we have analyzed gene-expression patterns and not protein levels. As gene-expression levels do not always fully represent the protein expression, this will need to be confirmed in further studies. However, we have previously shown in our model that pro-apoptotic proteins (cleaved caspase-3) are upregulated, leading to brain area loss at P14 (Osredkar et al., 2015). As we find a comparable level of brain area loss in our current study, we believe that pro-inflammatory and pro-apoptotic proteins will be involved, potentially activated by our pro-inflammatory gene-expression profiles. Second, we have only investigated early pro-inflammatory responses after 24 h yet. As M1/M2 phenotype expressions will change over time, we will need to analyze later time points. From a clinical perspective, new treatment interventions may only be applicable after understanding the complex time-dependent changes of inflammatory responses, as they might lead to early treatment interventions. This will be tested by us in the future and may lead to translational treatment options in newborns presenting with NE. Third, compared to our previous publications of this model (Osredkar et al., 2013, 2015), we did not find a significant increase of brain injury between the LPS/HI and Veh/HI groups. Pre-sensitization with LPS increased brain injury, compared to Veh/HI, without reaching the 5% significance level, most likely due to the mild HI injury in the current study compared to our previous publications. However, the pre-sensitization effect of LPS is clearly demonstrated by our results.

In summary, our results highlight that microglia are early key mediators of the inflammatory response following inflammation-sensitized HI brain injury, polarizing into a predominant pro-inflammatory phenotype 24 h post HI. Additionally, we demonstrate the involvement of the *NLRP3* inflammasome, possibly highlighting one potential regulatory pathway in our model. These findings will help us to better understand the complex pathophysiological changes in our model and in the future, this may give us the possibility to early intervene and offer new treatment options, helping to further improve outcome in asphyxiated newborns, especially in countries with high perinatal infection and perinatal asphyxia rates.

## Data Availability

All datasets generated for this study are included in the manuscript and/or the supplementary files.

## Ethics Statement

This study was carried out in accordance with the recommendations of the State Agency for Nature, Environment and Consumer Protection North Rhine-Westphalia, Germany. The protocol was approved by the State Agency for Nature, Environment and Consumer Protection North Rhine-Westphalia, Germany.

## Author Contributions

MS and HS planned and designed the study. MS and HS performed the animal experiments. MS, KK, MR, JH, IB, and HS performed tissue analysis. MS and HS analyzed the data. MS, JH, IB, and UF-M wrote and corrected the manuscript.

## Conflict of Interest Statement

The authors declare that the research was conducted in the absence of any commercial or financial relationships that could be construed as a potential conflict of interest. The reviewer MT declared a past co-authorship with one of the authors HS to the handling Editor.
